# Enfermedades crónicas en población afectada por el conflicto armado en Colombia, 2015

**DOI:** 10.26633/RPSP.2017.144

**Published:** 2017-11-07

**Authors:** Carlos Gómez-Restrepo, Carlos Javier Rincón, Mauricio Medina-Rico

**Affiliations:** 1 Pontificia Universidad Javeriana Pontificia Universidad Javeriana Bogotá Colombia Pontificia Universidad Javeriana. Bogotá, Colombia.

**Keywords:** Enfermedad crónica, violencia, vulnerabilidad en salud, vulnerabilidad social, Colombia, Chronic disease, violence, health vulnerability, social vulnerability, Colombia, Doença crónica, violencia, vulnerabilidade em saúde, vulnerabilidade social, Colombia

## Abstract

**Objetivo.:**

*El propósito de este estudio fue identificar las enfermedades crónicas no mentales, más frecuentes en la población colombiana afectada por el conflicto armado*.

**Metodología.:**

*Este es un estudio transversal, que usa datos de la Encuesta Nacional de Salud Mental 2015. La población estudiada se estratificó por sexo y edad y se extrajeron otros datos generales como: nivel educativo y grado de pobreza, medido por el Índice Multidimensional de Pobreza. Se analizaron datos de personas que reportaron haber sido víctimas del conflicto armado colombiano en algún momento de la vida la vida y se reportó como medida de riesgo relativo indirecto la frecuencia de presentación de enfermedades crónicas no mentales*.

**Resultados.:**

Se describió información de 10 764 personas mayores de 18 años siendo una muestra representativa a nivel nacional. Se encontró que 10,4 % de los sujetos afectados por el conflicto armado tienen un nivel educativo superior (técnico o universitario), así como 43,6 % se encuentran en estado de pobreza o en estado de vulnerabilidad. Las enfermedades crónicas no mentales identificadas fueron: hipertensión arterial 20,4 % (IC95% 15,7–26,1), diabetes 6,7 % (IC95% 4,4–10,3), enfermedades reumatológicas 10,4 % (IC95% 7,1–14,9), enfermedades gastrointestinales 19,1 % (IC95% 14,5–24,7), y dolor crónico 6,9 % (IC95% 4,2–11)

**Conclusiones.:**

*La población afectada por el conflicto armado tiene aparentemente mayor riesgo de presentar enfermedades crónicas no mentales, tales como hipertensión arterial y diabetes, lo que evidencia la situación de vulnerabilidad de estas comunidades*.

## Abstract (en)

***Objective*.:**

The purpose of this study was to identify the most frequent non-mental chronic illnesses in the Colombian population affected by the armed conflict.

**Methodology.:**

A cross-sectional study using data from the National Mental Health Survey 2015. The study population was stratified by sex and age and other general data were extracted, including education and poverty level, measured by the Multidimensional Poverty Index. Data analysis was based on information from people who reported having been victims of the Colombian armed conflict at some time in their lives; the frequency of presentation of non-mental chronic illnesses was reported as a measure of indirect relative risk.

***Results*.:**

Information on 10,764 people over 18 years of age was described, this being a representative sample at the national level. It was found that 10.4% of subjects affected by the armed conflict have a high educational level (technical school or university), and that 43.6% are living in conditions of poverty or vulnerability. The non-mental chronic illnesses identified were: hypertension 20.4% (CI95%: 15.7-26.1); diabetes 6.7% (CI95%: 4.4-10.3); rheumatologic diseases 10.4% (CI95%: 7.1-14.9); gastrointestinal diseases 19.1% (CI95%: 14.5-24.7); and chronic pain 6.9% (CI95%: 4.2-11)

**Conclusions.:**

The population affected by the armed conflict is apparently at greater risk of presenting non-mental chronic illnesses such as hypertension and diabetes, which shows the vulnerability of these communities.

## RESUMO

Identificar as doenças crônicas não relacionadas à saúde mental mais comuns na população oriunda de áreas de conflito armado.

**Metodologia.:**

Estudo transversal conduzido com dados da Pesquisa Nacional de Saúde Mental realizada na Colômbia em 2015. A população estudada foi estratificada por sexo e idade. Foram também coletadas informações gerais como nível de escolaridade e nível de pobreza, avaliado pelo Índice Multidimensional de Pobreza. Foram analisados dados de pessoas que se declararam vítimas do conflito armado em algum momento da vida e a frequência de ocorrência de doenças crónicas não relacionadas à saúde mental foi usada como medida de risco relativo indireto.

Foram obtidas informações de 10.764 pessoas acima de 18 anos de idade, constituindo uma amostra representativa ao nível nacional. Verificou-se que 10,4% das pessoas oriundas de áreas de conflito armado têm nível de escolaridade superior (técnico ou universitário) e 43,6% vivem em situação de pobreza ou vulnerabilidade. As doenças crônicas não relacionadas à saúde mental identificadas foram: hipertensão arterial (20,4%, IC95% 15,7–26,1), diabetes (6,7%, IC95% 4,4–10,3), doenças reumatológicas (10,4%, IC95% 7,1–14,9), doenças gastrointestinais (19,1%, IC95% 14,5– 24,7) e dor crônica (6,9%, IC95% 4,2–11).

**Conclusões.:**

A população oriunda de áreas de conflito armado aparentemente tem maior risco de apresentar doenças crônicas não relacionadas à saúde mental, como hipertensão arterial e diabetes, o que evidencia a situação de vulnerabilidade em que vivem estas comunidades.

Las enfermedades crónicas no transmisibles han sido la principal causa de muerte en la población adulta, teniendo un impacto diferente en países de alto y bajo ingreso siendo estas, potencialmente prevenibles (1). Un factor de riesgo que ha llamado la atención es la presencia de eventos traumáticos en la vida; principalmente aquellos relacionados con la violencia generada por conflictos armados y los efectos que estos conllevan, como es el caso de los desplazamientos, que afectan la posibilidad de continuar con la educación de los sujetos, dificultan el acceso a servicios de salud haciéndose estos más limitados y deficientes (2), además pueden favorecer el aumento de los trastornos mentales (3); estas condiciones también se asocian con la aparición temprana y/o empeoramiento de diferentes enfermedades crónicas (4, 5).

En las últimas cinco décadas, Colombia ha estado afectada por un constante conflicto armado interno, que entre los años 1985 y 2012 causó aproximadamente 220 000 muertes, siendo la mayoría víctimas civiles (6); Para el 7 de julio de 2016 el Registro Único de Víctimas en Colombia contaba con un total de 6 419 819 víctimas de desplazamiento (7).

El impacto que tiene en la salud factores promotores de enfermedades crónicas por conflictos armados se representa de manera diferente en las poblaciones pediátrica y adulta. En niños y adolescentes se documenta la aparición de estas enfermedades a edades más precoces (2), mientras que en adultos se percibe como la alteración de la calidad de salud (8, 9), secundario al malestar psicológico y alteraciones funcionales que ocasionen dichas enfermedades (10).

Algunos de los determinantes sociales que se alteran con más frecuencia en las poblaciones afectadas por conflictos armados son: percepción de abandono social; mayor riesgo de violencia intrafamiliar, abuso sexual, consumo de alcohol y otras sustancias psicoactivas; menor escolaridad; aumento de desempleo; y de pobreza (11). Igualmente, se ha encontrado en poblaciones desplazadas un mayor compromiso de la locomoción y del nivel sensorial (visual y/o auditivo), que se asocian a un menor bienestar en estos individuos (12). Todos los factores anteriores se relacionan con alteraciones de la salud física y emocional de las personas. Algunas de las condiciones crónicas que más se han encontrado asociadas al contacto con conflictos armados y otros eventos traumáticos son: La hipertensión (HTA), la enfermedad pulmonar obstructiva crónica (EPOC), la diabetes y cáncer (13, 14)

Según la Encuesta Nacional de Salud Mental realizada en Colombia en el año 2015 (ENSM-15) 7,9 % de la población adulta ha tenido que experimentar eventos traumáticos relacionados con el conflicto armado (15). Tales sucesos han dado como resultado el deterioro de la calidad de vida de las comunidades así como alteraciones de salud física y mental de la población Colombiana (16, 17). Sin embargo, esta situación no ha sido estudiada en profundidad en las encuestas de salud mental de 1993, 1997 y 2003. El objetivo del presente estudio fue identificar las enfermedades crónicas no mentales más frecuentemente reportadas en la población colombiana, que se ha visto afectada por el conflicto armado, mediante la información recogida en la ENSM-2015 de una muestra representativa de la población general.

## MÉTODOS

Este es un estudio observacional de corte transversal que tuvo como base una submuestra de la muestra maestra recolectada a partir de la Encuesta Nacional de Salud Mental Colombia 2015 (18). La muestra fue de tipo probabilístico, estratificada por sexo, edad (de 18 a 44 años y más de 45 años), estado civil, nivel educativo, y por las cinco regiones del país (Atlántica, Oriental, Central, Pacífica y Bogotá); diferenciando las personas residentes en hogares de áreas urbanas y rurales del país. Se analizaron los datos de los individuos que reportaron haber sido víctimas del conflicto armado alguna vez en la vida. Los criterios de exclusión fueron: sujetos con demencia, tener alguna limitación auditiva, visual o de lenguaje que impidiera responder la encuesta, no hablar español, personas institucionalizadas o que no desearan participar y no firmaran el consentimiento informado. Dado que este estudio toma los valores de la ENSM-2015, no se excluyeron personas con otros tipos de trastornos mentales pues algunos de estos fueron evaluados en la misma encuesta.

El estudio se realizó mediante una entrevista apoyada en un formato de aplicación digital. Para la selección de los instrumentos mencionados se realizó una búsqueda en la literatura de las escalas que se utilizan a nivel nacional e internacional y se determinó su aplicabilidad acorde a las necesidades requeridas, construyendose nuevos instrumentos basados en cuestionarios existentes bajo la supervisión de expertos temáticos en la elaboración de escalas.

Además de los datos generales del individuo y del hogar, se indagó el Índice de Pobreza Multidimensional (IPM), un indicador que refleja la privación de los hogares en cinco dimensiones relacionadas con: 1) condiciones educativas del hogar, 2) condiciones de la niñez y juventud, 3) trabajo, 4) salud y 5) acceso a servicios públicos domiciliarios y de la vivienda, pero que no incluye pobreza monetaria (19, 20). Para preguntar sobre la presencia de eventos violentos asociados o no al conflicto armado se realizó un cuestionario basado en preguntas utilizadas en la encuesta nacional de salud mental 2003 (21). Por otro lado para el diagnóstico de condiciones crónicas se elaboró un cuestionario en el cual se preguntó a los sujetos si estos habían sido diagnosticado por un médico con alguna enfermedad crónica según una lista de enfermedades que incluían: hipertensión arterial (HTA), diabetes, cáncer, falla renal, enfermedades tiroideas, enfermedades pulmonares como asma o enfermedad obstructiva crónica (EPOC), enfermedades cardiacas, gastritis, intestino irritable, enfermedades reumatológicas, enfermedades hepáticas, enfermedad cerebrovascular, dolor crónico, epilepsia u otras enfermedades crónicas.

### Análisis estadístico

Se estimaron porcentajes e intervalos con un nivel de confianza del 95 % para cada una de las variables bajo estudio, utilizando el método de linealización mediante series de Taylor para la estimación de la varianza en encuestas complejas utilizando STATA 14 (22, 23). Para este reporte se presentan los coeficientes de variación (CV) menores 33,3 % lo que indica que son estimaciones confiables estadísticamente y aquellos mayores a 33,3 % se marcan con un asterisco y se consideran imprecisos. Los resultados se reportaron a través del Riesgo Relativo Indirecto con su respectivos intervalos de confianza del 95 %.

Este estudio fue aprobado por el comité de ética de la Universidad Javeriana y el Ministerio de Salud de la República de Colombia, quienes dieron el aval para la realización de la Encuesta Nacional de Salud Mental 2015. Toda la información de los individuos encuestados es completamente confidencial asegurando el anonimato de los participantes.

## RESULTADOS

Los resultados de las 10 764 personas son considerados representativos de la población colombiana de 18 años en adelante. Respecto el nivel educativo el 14,7 % de los sujetos tienen un nivel educativo superior ya sea técnico o universitario. Por otro lado, para el índice de pobreza llama la atención que el 43,6 % de la población afectada por el conflicto armado se encontraban en estado de pobreza o en estado de vulnerabilidad. (cuadro 1)

Entre los hallazgos más evidentes se encuentra que la mayoría de enfermedades crónicas no mentales, son más frecuentes en la población en contacto con el conflicto armado y/o con antecedente de algún evento traumático relacionado o no con el conflicto armado, respecto las personas que nunca tuvieron eventos traumáticos ni contacto con el conflicto armado. (cuadro 2)

**CUADRO 1.** Características generales de la muestra en el estudio de enfermedades crónicas en población afectada por el conflicto armado en Colombia, 2015.

Características	Conflicto Armado		Conflicto Armado y otro evento traumático		Evento traumático distinto de Conflicto Armado		Ningún evento traumático		Total	
	n	%	n	%	n	%	n	%	n	%
Sexo										
Hombre	210	42,6	154	44,5	1 383	39,8	2 575	39,9	4 322	40,2
Mujer	283	57,4	192	55,5	2 095	60,2	3 872	60,1	6 442	59,8
Edad										
18 a 44	248	50,3	205	59,2	1 868	53,7	3 494	54,2	5 815	54,0
45 o más	245	49,7	141	40,8	1 610	46,3	2 953	45,8	4 949	46,0
Estado Civil										
Casado-Unión Libre-pareja	292	59,2	200	57,8	1 854	53,3	3 614	56,1	5 960	55,4
Separado/Viudo/divorciado	89	18,1	68	19,7	671	19,3	1 115	17,3	1 943	18,1
Soltero	112	22,7	78	22,5	953	27,4	1 718	26,6	2 861	26,6
Nivel Educativo										
Ninguno/Primaria	255	52,1	128	37,5	1 077	31,3	2 533	39,6	3 993	37,4
Secundaria	183	37,4	158	46,3	1 764	51,3	3 000	46,9	5 105	47,9
Técnico/Tecnólogo	32	6,5	27	7,9	334	9,7	481	7,5	874	8,2
Universitario	19	3,9	28	8,2	265	7,7	382	6	694	6,5
Región										
Central	110	22,3	86	24,9	757	21,8	1 201	18,6	2 154	20,0
Atlántica	97	19,7	30	8,7	530	15,2	1 698	26,3	2 355	21,9
Bogotá	40	8,1	51	14,7	771	22,2	831	12,9	1 693	15,7
Oriental	142	28,8	100	28,9	719	20,7	1 585	24,6	2 546	23,7
Pacífica	104	21,1	79	22,8	701	20,2	1 132	17,6	2 016	18,7
Zona										
Urbano	310	62,9	235	67,9	2 841	81,7	4 798	74,4	8 184	76,0
Rural	183	37,1	111	32,1	637	18,3	1 649	25,6	2 580	24,0
Pobreza										
Hogares con acceso	46	9,3	53	15,3	724	20,8	1 039	16,1	1 862	17,3
Hogares no vulnerables a PMD	232	47,1	159	46	1 862	53,5	3 324	51,6	5 577	51,8
Hogares vulnerables a PMD	115	23,3	81	23,4	576	16,6	1 237	19,2	2 009	18,7
Hogares en estado de pobreza	100	20,3	53	15,3	316	9,1	847	13,1	1 316	12,2

**- PMD** = Pobreza multi dimensional

***Fuente:***Datos extraídos de los resultados de la Encuesta Nacional de Salud Mental 2015 (18)

Tres grupos de enfermedades crónicas no mentales: dolor crónico, enfermedades reumatológicas (artritis o fibromialgia) y enfermedades gastrointestinales (reflujo gastrointestinal, gastritis, o síndrome de intestino irritable), muestran mayor probabilidad de presentarse dependiendo del tipo de evento estresor, teniéndose de menor a mayor riesgo: no haber estado en contacto con eventos traumáticos o con el conflicto armado; haber tenido algún evento traumático no asociados al conflicto armado; haber estado en contacto con el conflicto armado; y finalmente haber tenido un evento traumático además del contacto con el conflicto armado. Un ejemplo de lo anterior son las enfermedades reumatológicas que tienen un riesgo del 5 % (IC95% 4,4 – 5,7) en individuos sin contacto con conflicto armado ni historia de eventos traumáticos; 6,5 % (IC95% 14,5 – 24,7) en sujetos con antecedente de evento traumático diferente a conflicto armado; 10,4 % (IC95% 7,1 – 14,9) de personas con contacto con conflicto armado, y 12,1 % (IC95% 6,9 – 20,3) en personas con contacto con conflicto armado más antecedente de algún evento traumático en la vida. Otras enfermedades crónicas que mostraron diferencia son: HTA 16 % (IC95% 14,8 – 17,4) en población sin conflicto y de 20,4 % (IC95% 15,7 – 26,1) en población con conflicto, y la diabetes de 5 % (IC95% 4,3 – 5,8) respecto un 6,7 % (IC95% 4,4 – 10,3).

Condiciones como las enfermedades renales, el EPOC, las enfermedades coronarias y los eventos cerebrovasculares fueron imprecisos en uno de los dos escenarios que contemplan el conflicto armado, por lo cual no es confiable hacer una adecuada descripción de los mismos. Así mismo el cáncer, las enfermedades tiroideas, las enfermedades hepáticas y la epilepsia fueron enfermedades que durante el análisis mostraron ser imprecisas para su comparación en los escenarios en que se tiene como estresor al conflicto armado.

## DISCUSIÓN

En este estudio se observa cómo algunas condiciones crónicas como la hipertensión arterial o la diabetes parecen ser más frecuentes en personas afectadas por el conflicto armado respecto la población general. De la misma manera llamó la atención cómo algunas enfermedades reumatológicas y gastrointestinales como dolor crónico y gastritis parecen ser más frecuentes en personas con historia de eventos traumáticos tanto asociados a conflicto armado como no asociados a estos.

Los hallazgos del estudio son similares a los encontrados en otros países que han tenido que sufrir los problemas que conllevan a conflictos armados. Doocy y cols. (24), identificaron la prevalencia de las condiciones crónicas en las poblaciones afectadas por el conflicto armado en Siria, siendo la hipertensión arterial y los problemas músculo esqueléticos las enfermedades más frecuentes con una prevalencia entre 17 y 20%, seguido de problemas gastrointestinales, diabetes y enfermedades cardiovasculares, con una prevalencia entre 7 y 11% (24). Por otro lado, en cuanto la presencia de eventos traumáticos no violentos Kessler y cols.(25) evaluaron la prevalencia y empeoramiento de las condiciones crónicas en poblaciones afectadas por catástrofes naturales, encontrando que estas son más altas que la población general, lo cual asocia a la interrupción del tratamiento médico secundario a un menor acceso tanto a medicamentos como a personal del área de la salud en dichas comunidades (25). Aunque existen estudios que han evaluado la violencia y su relación con enfermedades crónicas, como el de Suglia y cols. (26) que describen la asociación entre violencia y enfermedades cardiovasculares (26), o el de Georgey cols. (27) sobre la presencia de dolor crónico en víctimas de conflictos armados (27), pocos estudios existen que evalúen la incidencia de enfermedades crónicas en comunidades afectadas por conflictos armados y la incidencia de las mismas en situaciones antagónicas; bajo esta dificultad éste estudio puede servir como un posible precursor para futuras investigaciones sobre el tema.

La OMS en el 2005 describe dentro de la comisión de los determinantes sociales en salud la importancia de mejorar las condiciones de vida cotidiana, donde se menciona la importancia de la educación, equidad, empleo digno, atención en salud y disminución de la violencia (28); todas estas condiciones se afectan en las comunidades que enfrentan conflictos armados. Llama por ejemplo la atención, que una importante proporción de la población del estudio se encuentra en estado de pobreza o en estado de vulnerabilidad, lo cual se puede traducir en un acceso a servicios de salud deficiente (29). Este estudio colabora con la función de la OMS de medición y análisis del problema (28), comenzando por la descripción de la situación que se origina del conflicto armado y el impacto que este produce en el desarrollo de diferentes enfermedades crónicas por el distrés que nace de estos antecedentes.

Pese a que este estudio se encargó de observar la presencia de enfermedades crónicas no mentales en la población afectada por el conflicto armado; para el campo de la salud mental existen datos de la ENSM-2015 en que se midió la presencia de los diferentes trastornos mentales en estas comunidades (depresión, ansiedad, consumo de alcohol y otras sustancias psicoactivas principalmente marihuana ), los cuales ya han sido utilizados para el desarrollo de otros estudios que se enfocaron específicamente en la evaluación de la salud mental (30, 31).

En esta investigación no se consideró evaluar la relación entre trastornos mentales y condiciones crónicas, pues debido que al ser de corte transversal, el diseño no permitía evaluar una asociación entre tales hallazgos, no obstante, ante la relación entre enfermedades crónicas no mentales y conflictos armados, y enfermedades mentales y conflictos armados, nuestro estudio da unas bases para futuras investigaciones sobre la relación entre estos componentes de la salud que permitan crear conocimiento dirigido al desarrollo de intervenciones en estas comunidades, siendo esta una de las tareas tanto de la Organización Mundial de la Salud, como de la Organización Panamericana de la de Salud (32).

Pese a que los resultados encontrados parecen indicar una posible relación entre el contacto con el conflicto armado y el desarrollo de algunas enfermedades crónicas no mentales, nuevamente debido al diseño del estudio, no se puede asegurar una relación causal entre los datos de interés. No obstante, existe información acerca de los efectos nocivos que tiene la presencia de eventos adversos como: el maltrato físico, sexual y emocional sobre la salud física, principalmente cuando se presentan en la infancia o en la adolescencia, y que se manifiestan en la vida adulta en forma de enfermedades crónicas no mentales como: la HTA (33), el EPOC (14) y el cáncer (29). Siendo que el conflicto armado puede ir acompañado de los problemas antes mencionados, se hace evidente la necesidad de estudiar a mayor profundidad el impacto que tiene en la salud física el contacto con el conflicto armado, así como evaluar una forma de mejorar los mecanismos con los cuales se ha venido interviniendo a estas comunidades.

**CUADRO 2.** Presencia de enfermedades crónicas en individuos afectados por eventos traumáticos y conflicto armado. Linealización mediante series de Taylor para la estimación de la varianza en encuestas complejas para estimación de porcentajes e intervalos de confianza del 95 %

	Conflicto armado			Conflicto armado y otro evento traumático			Evento traumático distinto de conflicto armado			Ningún evento traumático			Total		
	%	IC95%		%	IC95%		%	IC95%		%	IC95%		%	IC95%	
Presión alta, tensión alta o hipertensión	20,4	15,7	26,1	18,7	12,2	27,6	16	14,3	18	16	14,8	17,4	16,3	15,3	17,3
Diabetes o azúcar alta en la sangre	6,7	4,4	10,3	6,4	3,4	11,9	5,5	4,3	7	5	4,3	5,8	5,3	4,6	5,9
Tumor maligno o cáncer	1,1^*^	0,5	2,8	0,5^*^	0,1	2,5	1,8	1,3	2,5	1,1	0,8	1,4	1,3	1,0	1,6
Falla renal u otra enfermedad que cause un daño crónico en el riñón	3,8	2,1	6,7	5,8^*^	2,5	12,8	2,9	2,3	3,7	1,9	1,5	2,4	2,4	2,1	2,9
Enfermedades de la tiroides como hipotiroidismo o hipertiroidismo	1,2^*^	0,5	3,2	2,2^*^	0,8	5,7	5,3	4,2	6,6	3,4	2,8	4,1	3,9	3,4	4,5
Enfermedades pulmonares como asma, bronquitis crónica, enfisema o enfermedad pulmonar obstructiva crónica	5,0	2,9	8,3	5,7^*^	2,6	12,2	5,1	4,1	6,2	3,7	2,9	4,7	4,3	3,7	5,0
Enfermedades del corazón como ataque al corazón, infarto cardiaco, enfermedad coronaria, angina o falla cardiaca	4,5	2,6	7,7	11,6^*^	5,3	23,4	4,2	3,3	5,2	3,0	2,4	3,8	3,7	3,2	4,4
Reflujo, gastritis, úlcera gástrica o intestino irritable	19,1	14,5	24,7	30,7	22,9	39,8	22,6	20,5	24,8	15,4	14	16,9	18,4	17,2	19,5
Enfermedad mental o de los nervios	4,1	2,3	7,4	3,7^*^	1,6	8,5	3,8	3	4,9	2,7	2,2	3,3	3,2	2,7	3,7
Artritis, reumatismo, artrosis, fibromialgia u otras enfermedades autoinmunes	10,4	7,1	14,9	12,1	6,9	20,3	6,5	5,3	7,9	5,0	4,4	5,7	5,9	5,3	6,6
Enfermedades que producen daño crónico en el hígado como hepatitis alcohólica, tóxica, por virus o de otra causa	0,7^*^	0,1	4,1	3,6^*^	1,3	9,6	2,1	1,4	3,1	0,5	0,3	0,6	1,1	0,8	1,5
Enfermedad cerebrovascular, trombosis o derrame cerebral	1,2^*^	0,5	3,4	0	-	-	1,0	0,6	1,6	0,5	0,3	0,7	0,6	0,5	0,9
Dolor crónicos	6,9	4,2	11	11,9	6,7	20,1	6,3	5,2	7,7	3,5	2,8	4,2	4,8	4,2	5,5
Epilepsia o convulsiones	0,5^*^	0,1	1,8	0,8^*^	0,2	2,5	0,8	0,5	1,2	0,6	0,4	0,9	0,6	0,5	0,9
Otra enfermedad crónica	1,4^*^	0,5	3,8	2^*^	0,9	4,5	3,2	2,4	4,3	1,6	1,2	2,1	2,1	1,7	2,6

* Estimaciones con coeficiente de variación mayor a 33%.

***-Fuente:*** Datos extraídos de los resultados de la Encuesta Nacional de Salud Mental 2015 (18)

En la actualidad Colombia se encuentra cursando un proceso de reconciliación y posconflicto y aunque no se cuenta con información para observar cómo han ido cambiando los datos en el último año, se espera en el futuro tener una mejoría de la salud general de los individuos que han tenido que vivir en las zonas de conflicto armado (34, 35).

Como fortalezas, el estudio tiene un diseño poblacional y aplicabilidad de una muestra probabilística representativa para el país. Igualmente, haber usado instrumentos para la clasificación de la exposición a violencia. Teniendo en cuenta en el diseño del estudio, el tipo de análisis propuesto y la poca información existente acerca de la asociación del conflicto armado con las condiciones crónicas no mentales, éste estudio constituye un insumo para profundizar en el tema a futuro, evaluando todo el espectro que abarca el conflicto armado y considerando posibles relaciones causales que eventualmente permitan diseñar estrategias puntuales de promoción y prevención en la salud de los sujetos.

Las limitaciones del estudio están dadas por el hecho de ser un estudio transversal, y pese a los objetivos planteados, no se pueden definir si hay una verdadera asociación o relación causal entre los datos de interés, y la modificación de algunos instrumentos limita la comparabilidad con otros estudios; no obstante, cabe precisar que la descripción del problema si bien no es suficiente, es el primer paso para incentivar y fundamentar futuras investigaciones con un diseño que permita indagar la posible asociación que aquí se plantea y cuyos resultados ayuden a la elaboración de políticas de salud pública.

 Nuestros datos sugieren que la proximidad con el conflicto armado y la presencia de eventos traumáticos a lo largo de la vida puede afectar la salud física de las personas relacionándose con una mayor frecuencia de condiciones crónicas no mentales tales como: la hipertensión arterial, la diabetes, las enfermedades pulmonares,gastrointestinales y reumatológicas. La relación entre estas variables es compleja, pudiendo existir otros aspectos a considerar como: tipo de exposición, intensidad y duración de los eventos, y las características culturales de la comunidad que pueden influir en la adaptación y capacidad de resiliencia de los sujetos. Se anima a otros investigadores a desarrollar estudios que permitan medir la asociación del conflicto armado y las enfermedades crónicas, y evaluar posibles estrategias a implementar desde la salud pública que contribuyan, a disminuir los efectos que tiene la violencia en la salud de las personas. Finalmente, Colombia, es un país con una historia de violencia que actualmente se encuentra en un proceso de diálogos con lo cual se espera mejorar la situación de violencia, no obstante, para ello se debe hacer el respectivo seguimiento en los próximos años y medir el impacto que esto tenga en el sistema de salud.

### Agradecimientos.

Al Ministerio de Salud y Protección Social quienes dieron su apoyo para la ejecución de la Encuesta Nacional de Salud Mental “ENSM”.

### Financiación.

La encuesta fue financiada por COLCIENCIAS y el Ministerio de Salud y Protección Social, contrato 762-2013. Bogotá, Colombia.

### Declaración.

Las opiniones expresadas en este manuscrito son responsabilidad del autor y no reflejan necesariamente los criterios ni la política de la *RPSP/PAJPH* y/o de la OPS
